# Xth EPR Workshop on Applications of EPR in Biology and Medicine

**DOI:** 10.1007/s12013-017-0826-1

**Published:** 2017-10-25

**Authors:** Tadeusz Sarna, Balaraman Kalyanaraman, Lawrence Berliner

**Affiliations:** 1 Laboratory of Photobiophysics, Faculty of Biochemistry, Biophysics and Biotechnology, Jagiellonian University, 7 Gronostajowa St., 30-387 Krakow, Poland; 2 Medical College of Wisconsin, 8701 Watertown Plank Road, Milwaukee, WI 53226-0509 USA; 3 Cell Biochemisrty and Biophysics, University of Denver, Denver, CO 80208 USA

We are pleased and honored to present this special double issue for CBBI on the broad topic of biomedical EPR. The papers herein resulted from the most recent EPR Workshop in Kraków that encompasses work from outstanding researchers in the field. Before describing the range of articles, we have briefly summarized the history of these workshops and the publications that resulted.

## Short History of EPR Workshops in Krakow

Professor Stanislaw Lukiewicz conceived and organized the first EPR Workshop in Krakow in 1989. From the onset these workshops have usually been dedicated to some special occasion, such as an anniversary or special scientific honor of a particular famous researcher. The idea to invite word-leading investigators in the EPR field to the Jagiellonian University in Krakow first came to his mind on the occasion of the conferment of Doctor Honoris Causa (honorable doctor) on Professor James Stewart Hyde, which took place in September 1989. The event was followed by a series of lectures by invited guests, which took the form of an informal workshop, allowing for discussions of common ideas, problems, and achievements, including experimental equipment and methods developed in the Krakow EPR laboratories. This was the first Workshop on Applications of EPR in Biology and Medicine (“EPR Workshop”), which proceeded the second in December 1992, organized also by Lukiewicz. The 3rd EPR Workshop was organized by Prof.Witold Karol Subczynski in September 1995, which celebrated the 25th Anniversary of the founding of the Institute of Molecular Biology, Jagiellonian University, that was followed by a separate Workshop on Nitric Oxide and Immune Responses to Allografts and Tumors (“NO­Workshop”), organized by Prof. Lukiewicz in November-December 1995, that comprised a substantial number of EPR-related contributions. The 4th EPR Workshop was organized in September 1998 by Prof. Tadeusz Sarna, honoring the 70th Birthday of Prof. Lukiewicz. The 5th EPR Workshop took place in 2001 and the American contingent buoyed everyone by coming in full force two weeks after the September 11th bombings, when the Krakow group was ready to call off the event. It was devoted to the opening of the Institute of Biophysics Molecular Biology and Biotechnology in a new building in Krakow–Pychowice. Next the 6th EPR Workshop was organized in October 2004 by Prof. Tadeusz Sarna, Prof. Wojciech Froncisz, and Prof. Balaraman Kalyanaraman (Milwaukee), which was the last Workshop with the participation of Prof.Lukiewicz before he died. The 7th and 8th EPR Workshops (October 2007 and 2010), the latter one coinciding with the 40th Anniversary of the founding of the Faculty) were co-organized by Profs.Tadeusz Sarna and Balaraman Kalyanaraman. The 8th EPR Workshop included a special session dedicated to late Ted Walczak. The Chairs of the 9th EPR 2013 Workshop were Professors Sarna, Kalyanaraman, and Wolfgang Lubitz from the Max Planck Institute. And subsequently, in 2016, the Xth EPR Workshop was organized for the 10th time, jointly by Profs Sarna, Kalyanaraman and Bruce Freeman from the University of Pittsburgh.

Some of the Workshops were followed by special EPR issues of the Journal of the Polish Biophysical Society-Current Topics in Biophysics. The 1st issue was published in 1994, the 2nd in 1996, the 3rd in 1999, the 4th in 2002, and the 5th in 2005. The Nitric Oxide Workshop was followed with a book “Nitric Oxide in Allograft Rejection and Anti-Tumor Response,” coedited by Prof. Jay L Zweier and Prof. Stanislaw J. Lukiewicz, published by Kluwer Academic Publishers in 1998. It was then awarded the Prize of the King’s Town of Krakow in 1999, acknowledging the promotion of Krakow by all of these workshops organized by the Jagiellonian University.

## Layout of the Special Issue

We have broken down the various papers into five general topic areas**:** EPR instrumentation, EPR oximetry, oxidative stress (redox status, reactive oxygen species), photosynthesis and photo related systems, membranes (structure and radical chemistry).

EPR instrumentation is elegantly covered by Jim Hyde and colleagues on W-band rapid frequency sweeping and Caston and colleagues describes implantable resonator development.

EPR oximetry is covered in the papers by Kuppusamy and colleagues on cerebral ischemia, Halpern and colleagues on pO2 imaging and Gustafsson and colleagues on head and neck tumors.

Under the oxidative stress subtopic, Kalyanaraman and colleagues present a paper on modified metformin effects; this is followed by a paper by Zadlo and colleagues relating to aspects of melanin redox chemistry; J Zielonka and colleagues talk about NADPH oxidase; Marakova and colleagues present a paper on bisphenol A degradation; Kruczała and colleagues look at free radicals in the blood; and Lohan and colleagues describe processes in keratinocytes

Under membranes (structure and radical chemistry); Subczynski and colleagues covers cholesterol: Effects in biological membranes; Widomskaas and colleagues describe bilayer domains in the eye lens; Kusumi and colleagues cover GPI-anchored receptors: Girotti and colleagues discusses cholesterol hydroperoxide generation.

Lastly, for photosynthesis and photo related systems Tikhonov describes electron in proton transport in chloroplasts; Anna Wisniewska–Becker and colleagues cover model photoreceptor membranes; and Pawlak and colleagues describe photoreactivity of bovine retinal lipids.

The editors hope that you enjoy this double issue and learn about the impressive progress that has occurred to date.

